# Comparison of the effects of Mitomycin-C and sodium hyaluronate/carboxymethylcellulose [NH/CMC] (Seprafilm) on abdominal adhesions

**DOI:** 10.1186/s40064-016-2359-2

**Published:** 2016-06-23

**Authors:** Ismail Hakkı Ozerhan, Murat Urkan, Ulvi Mehmet Meral, Aytekin Unlu, Nail Ersöz, Funda Demirag, Gokhan Yagci

**Affiliations:** Departments of Surgery, Gulhane Military Medical Academy, 06018 Etlik, Ankara Turkey; Department of Pathology, Atatürk Chest Diseases and Chest Surgery Education and Research Hospital, Ankara, Turkey; Departments of Surgery, Ankara Guven Hospital, Ankara, Turkey

## Abstract

**Introduction:**

Intra-abdominal adhesions (IA) may occur after abdominal surgery and also may lead to complications such as infertility, intestinal obstruction and chronic pain. The aim of this study was to compare the effects of Mitomycin-C (MM-C) and sodium hyaluronate/carboxymethylcellulose [NH/CMC] on abdominal adhesions in a cecal abrasion model and to investigate the toxicity of MM-C on complete blood count (CBC) and bone marrow analyses.

**Methods:**

The study comprised forty rats in four groups (Control, Sham, Cecal abrasion + MM-C, and Cecal abrasion + NH/CMC). On postoperative day 21, all rats except for the control (CBC + femur resection) group, were sacrificed. Macroscopical and histopathological evaluations of abdominal adhesions were performed. In order to elucidate the side effects of MM-C; CBC analyses and femur resections were performed to examine bone marrow cellularity.

**Results:**

CBC analyses and bone marrow cellularity assessment revealed no statistically significant differences between MM-C, NH/CMC and control groups. No significant differences in inflammation scores were observed between the groups. The MM-C group had significantly lower fibrosis scores compared to the NH/CMC and sham groups. Although the adhesion scores were lower in the MM-C group, the differences were not statistically significant.

**Conclusion:**

Despite its potential for systemic toxicity, MM-C may show some anti-fibrosis and anti-adhesive effects. MM-C is a promising agent for the prevention of IAs, and as such, further trials are warranted to study efficacy.

## Background

Intraabdominal adhesions (IAs) are among the leading causes of postoperative complications (Celebioglu et al. 1999). IAs may occur due to surgical technique, trauma, foreign bodies (fibrin glue and oxidized-regenerated cellulose, patches, meshes, glove powder) and surgical sutures. Postoperative IAs have a significant impact on morbidity (chronic pain, infertility, partial or complete small bowel occlusion, etc.) rates and also increase the workload in surgical units (Celebioglu et al. 1999; Sulaiman et al. 2001; Brüggmann et al. 2010). It has been reported that up to 100 % of patients develop IA after surgical interventions (Sulaiman et al. 2001; Brüggmann et al. 2010). These patients frequently require a secondary adhesiolysis procedure and 8–32 % of these patients develop recurrent obstruction after the initial adhesiolysis procedure (Ellis 1997).

In view of the magnitude of the health problems and financial burden related to adhesions, prevention or reduction of postoperative adhesions has become an important priority (Holmdahl and Risberg 1997; Schnüriger et al. 2011). Thus, prevention of IAs have been addressed by numerous experimental studies, although with limited success rates (Kamel 2010; Maciver et al. 2011).

In an attempt to attain improved outcomes the use of good surgical technique and anti-adhesion adjuvants (Adept^®^, Interceed^®^, Seprafilm^®^, etc.) have been introduced. Seprafilm^®^ (sodium hyaluronate/carboxymethylcellulose [NH/CMC], Genzyme Biosurgery Corporation, Cambridge, MA, USA) is the most widely studied adjuvant for the prevention of adhesions (Gonzales-Quintero and Cruz-Pachano 2009). NH/CMC has been reported to be highly effective in reducing both the incidence and severity of polypropylene mesh related adhesions (Dinsmore et al. 2000). Moreover, the anti-adhesive affect of NH/CMC was not diminished by the presence of visceral trauma and the resultant inflammatory response (Kumar et al. 2009).

Mitomycin-C (MM-C) (Mitomycin-C^®^ Kyowa Hakko Kogyo. Co. Ltd, Ohtemachi, Chiyoda-ku, Tokyo, Japan) is a promising agent that possess antiproliferative properties. It is an antineoplastic antibiotic that alkylates and crosslinks DNA (Kaufman et al. 2013). Local application of 0.02 % MM-C in topical eye solutions has been widely used in strabismus surgery to limit postoperative adhesions in humans, in dacryocystorhinostomy to prevent the obstruction of common canaliculus (Cheng et al. 2013; Cano-Parra et al. 1995; Mahindrakar et al. 2001) and also to prevent pterygia recurrence after excision in rabbits (Minguini et al. 2000). Moreover, intraperitoneal administration of MM-C was found to be effective and safe for the prevention of primary or recurrent IAs in rats (Cubukçu et al. 2001).

In this experimental study, we aimed to substantiate the individual affects of NH/CMC and MM-C for reducing IAs in a rat cecal abrasion model. We hypothesized that the two agents would significantly reduce IAs. We also investigated the toxicity of MM-C by complete blood count (CBC) analysis and examination of rat bone marrow specimens in the groups that received MM-C and in the control group.

## Methods

The experimental study was approved by the Animal Care and Use Ethics Committee of Gulhane Military Medical Academy. All animals received humane care in accordance with the Guide for the Care and Use of Laboratory Animals published by the National Institute of Health. Wistar albino female rats, weighing from 250 to 300 g, were used in this experimental study. All rats were quarantined for 1 week prior to the onset of study. All surgical interventions were carried out under general anesthesia using intramuscular 40 mg/kg ketamine hydrochloride (Ketalar^®^, Parke-Davis/Eczacıbası, Turkey) and 6 mg/kg Xylazine hydrochloride (Rompun^®^, Bayer, Mefar, Turkey). Sterile surgical technique was used throughout the study.

During the study design, authors assumed that the MM-C group would show inflammation and fibrosis scores of 3 and adhesion score of 4 in at least 10 % of rats. In comparison, sham group inflammation and fibrosis scores would be 3 and adhesion score would 4 in at least 60 % of rats. Thus, the sample size for attaining an alfa error of 0.05 and a beta error of 0.20 would require at least 10 rats per group. Accordingly, forty Wistar albino female rats were randomly and evenly assigned into four study groups. In order to elucidate the potential toxic effects of MM-C and decrease possible confounding factors, Group 1 (control) was created and we analyzed the basal values CBC counts and bone marrow morphologies. Except for Group 1, all other study groups were given a laparotomy with a 3 cm midline incision. Afterwards, the cecum was exteriorized with approximately 1 cm^2^ of its antero-medial serosal layer denuded by brushing ten times with a sterile toothbrush. Group 2 (sham) was administered 5 ml of saline solution intraperitoneally. Group 3 was administered 1 mg/kg of MM-C in 5 ml of saline solution, intraperitoneally. In Group 4, a 1 × 1 cm NH/CMC sheet was directly applied on the abrasion area. The cecum was then returned to the abdominal cavity and the abdomen was closed with continuous 4/0 silk sutures.

**Fig. 1:**
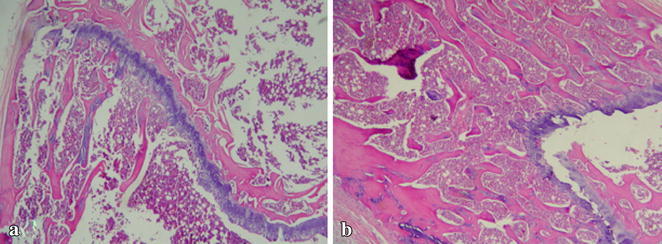
Bone marrow from control group (**a**) showing normal marrow cells (HEX40) Bone marrow from MMC group (**b**) resembles control group (HEX40)

On the 21st day, the rats were anesthesized and a second laparotomy was performed through a U-shaped incision for optimum exposure. The adhesions were graded by a blinded surgeon using the criteria described by Nair et al. (1974) (Table 1; Fig. 1). Rats with adhesion grades of 0 and 1 were considered adhesion free or insubstantial, grades between 2 and 4 were considered substantial or significant. Visceral and parietal tissues were resected and fixed in a 10 % formalin solution for at least 24 h by a blinded pathologist. Pathological examination of the specimens with adhesions were graded according to the presence of fibrosis and inflammation using a semiquantitative scoring system (Hooker et al. 1999) (Tables 2, 3).

**Table 1:** Nair et al.’s grading criteria for adhesions in rats

Grade	Description	Classification
0	Complete absence of adhesions	Insignificant adhesions insubstantial
1	Single band of adhesions between viscera or from one viscus to abdominal wall	Insignificant adhesions insubstantial
2	Two bands, either between viscera or from viscera to abdominal wall	Significant adhesions substantial
3	More than two bands between viscera or viscera to abdominal wall or whole of intestines forming a mass without being adherent to the abdominal wall	Significant adhesions substantial
4	Viscera directly adherent to abdominal wall regardless of number or extent of adhesive bands	Significant adhesions substantial

**Table 2:** Fibrosis grades

Score	Microscopic changes
0	Null
1	Minimal, loose
2	Moderate
3	Florid, dense

**Table 3:** Inflammation grades

Score	Microscopic changes
0	Null
1	Giant cells, occasional lymphocytes, and plasma cells
2	Giant cells, plasma cells, eosinophils, neutrophils
3	Many inflammatory cells, microabscesses

Blood for CBC analysis was aspirated via caval puncture during rat scarification. Then the femurs of rats (in MM-C and control groups) were amputated for bone marrow examination. Bones were decalcified using Shandon™ TBD-1™ Decalcifier (Thermo Scientific™, USA). These tissues were embedded in paraffin blocks and cut into 6 μm sections. The sections were stained with haematoxylin–eosin for bone marrow cellularity. Bone marrow cellularity assessment was performed by the blinded pathologist.

Statistical analysis was performed using SPSS Version 15.0 (SPSS Inc., Chicago, IL, USA). Kruskal–Wallis test was used to compare continuous variables. Bonferroni adjusted Mann–Whitney *U* and Dunnett’s multiple comparison test were used for comparing each group. Pearson Chi square test was used for comparisons of categorical variables. Statistical significance was set at 0.05.

## Results

None of the rats died as a result of anesthesia or in the follow-up period. There were no surgical wound related complications in any of the study groups. There were no statistically significant differences in white blood cell, neutrophil, red blood cell, hemoglobin, hematocrit, platelet counts between the control and other groups (p > 0.05). The control and MM-C group bone marrow cellularities were similar and assessed histologically as normocellular (Fig. 1a, b).

Inflammation scores were distributed between 0 and 1 in 70 % of the study groups and there were no statistically significant differences in inflammation scores between the study groups (p = 0.47). Analysis of fibrosis scores in the MMC group revealed that 100 % of scores were distributed between 0 and 1. Moreover, 70 % of Seprafilm and 80 % of sham group fibrosis scores were distributed between 2 and 3, respectively. Statistical analysis showed that MMC group fibrosis scores were significantly lower than the Seprafilm and Sham group scores (p > 0.001). Interestingly, there was no statistically significant difference between the Seprafilm and Sham group fibrosis scores (p > 0.05).

**Fig. 2:**
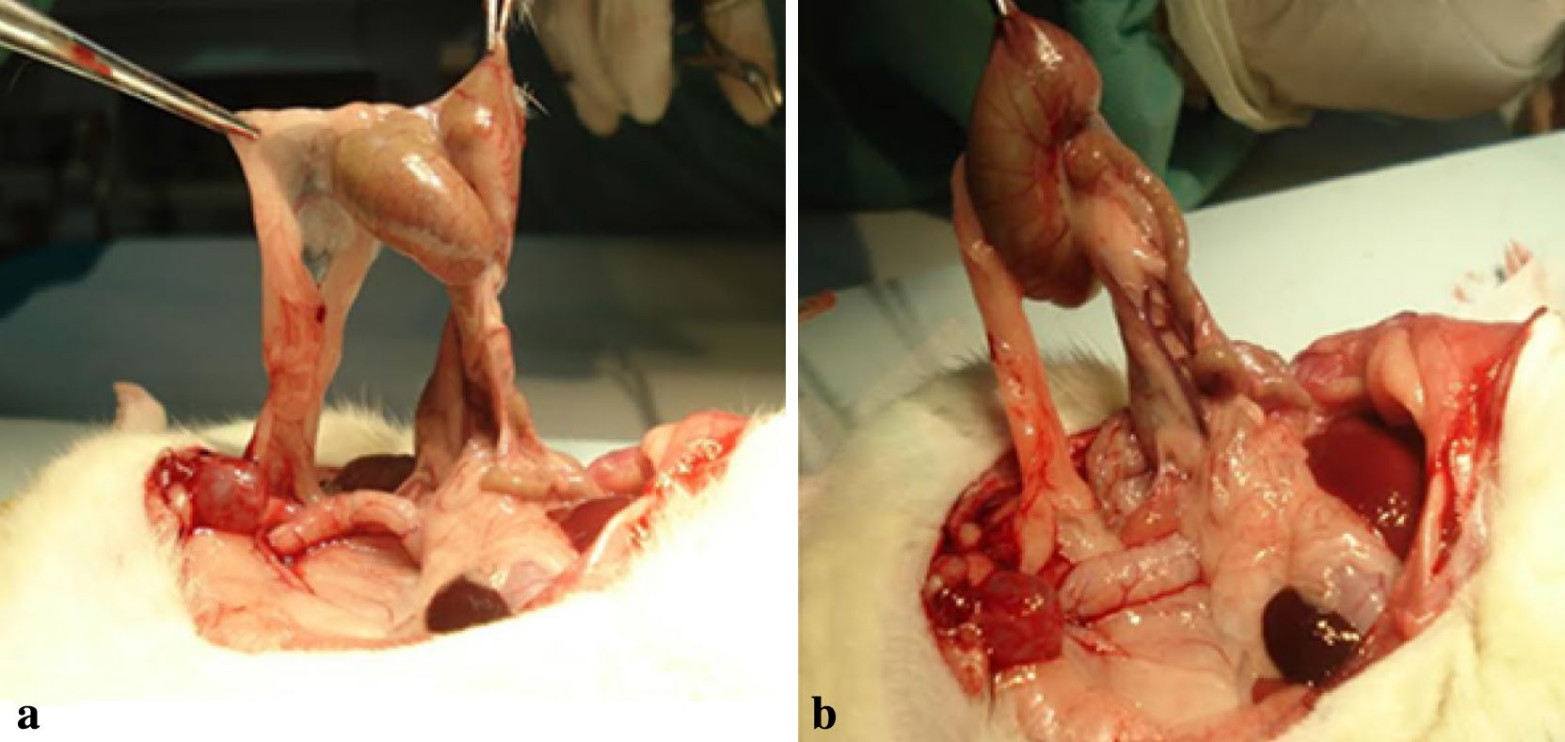
Exemplary peritoneal adhesion after 21 days in the sham group from rat 4 (**a**) and rat 6 (**b**)

The authors observed that the MM-C group had more insubstantial adhesions than the other groups. This was despite the fact that 70 % of the adhesion scores were scored as 0 and 1 in the MM-C group, 80 % as 1 and 2 in the Seprafilm group, and 80 % as 2 and 3 in the NaCl group, however, the differences were not statistically significant (p = 0.22) (Table 4; Fig. 2a, b).

**Table 4:** Distribution of inflammation, fibrosis and adhesion scores between study groups

Parameter	MM-C n (%)	Seprafilm^®^ n (%)	Sham n (%)	p value
*Inflammation score*				
0	5 (50 %)	4 (40 %)	5 (50 %)	0.473
1	2 (20 %)	3 (30 %)	2 (20 %)	
2	3 (30 %)	1 (10 %)	3 (30 %)	
3	0	2 (20 %)	0	
*Fibrosis score*				
0	6 (60 %)	2 (20 %)	1 (10 %)	0.018^a^
1	4 (40 %)	1 (10 %)	1 (10 %)	
2	0	2 (20 %)	2 (20 %)	
3	0	5 (50 %)	6 (60 %)	
*Adhesion score*				
0	4 (40 %)	2 (20 %)	1 (10 %)	0.224
1	3 (30 %)	2 (20 %)	1 (10 %)	
2	3 (30 %)	6 (60 %)	6 (60 %)	
3	0	0	2 (20 %)	
4	0	0	0	

^a^Statistically significant (*p* < 0.05)

## Discussion

Adhesions gather tissues and organs that are not normally associated (Ahmad et al. 2014). Incidence rates for abdominal adhesions have been estimated to be as high as 95 % after laparotomies (Attard and MacLean 2007). The process of IAs may begin within a few hours after surgery, and most commonly (60–70 %) present as small bowel obstruction (SBO) (Catena et al. 2011). SBOs represent an important cause of hospital admissions that generate a substantial burden on healthcare systems worldwide and they are frequently associated with significant mortality and morbidity rates. Simple intestinal obstructions and bowel necrosis/perforations related mortality rates have been reported as 3 and 30 %, respectively (Catena et al. 2008). Additionally, numerous studies have also shown that pelvic adhesion is frequently associated with chronic pelvic pain and infertility (Rajab et al. 2010; Howard 2000). The presence of IAs during repeated surgeries may increase the duration of surgery and also increase intraoperative complications, including damage to the intestines, bladder, ureters, and bleeding (Cheong et al. 2001). Even more drastically, 10- and 30-year SBO recurrence rates were 18 and 29 %, respectively, after an initial adhesiolysis procedure (Catena et al. 2008).

Collagen deposition in normal wound healing reaches a peak by the third week after the wound is created. After the third week, the wound undergoes constant alterations, known as remodeling, which can last for years after the initial occurrence of the injury (Mercandetti et al. 1298). Thus, the current study was designed to sacrifice all rats on the 21st day in order to appropriately determine the extent of adhesions.

Although the exact pathological mechanisms that underly the formation of IAs have not been fully elucidated; tissue injury, ischemia, infection and foreign bodies are among the foremost factors that induce fibrin deposition (Zhou et al. 2007). Fibrin deposition is due to an imbalance between the fibrin-forming and fibrin-dissolving capacities of a peritoneum, which results in the formation of post-surgical adhesions (Ersoy et al. 2009). In order to establish effective treatment protocols that prevent adhesion formation, a better understanding of the pathogenesis of IAs is required. Despite the fact that various agents (interleukins, corticosteroids, nonsteroidal anti-inflammatory drugs, lactated Ringer’s solution, dextran, etc.) to prevent postsurgical adhesion formation have been evaluated, none of these studied agents have proven successful (Numanoğlu et al. 2007; Parsaei et al. 2013).

Theoretically, inert materials that form a barrier and prevent contact between the damaged serosal surfaces for the first few critical days may allow separate healing of injured surfaces and help in the prevention of adhesion formation (Schnüriger et al. 2011). One example of such material is Seprafilm, which is composed of hyaluronic acid with carboxymethylcellulose. It turns into a hydrophilic gel 24 h after placement and provides a protective coat for traumatized tissues for up to 7 days (Kamel 2010).

MMC is another promising agent, and is an antibiotic isolated from a *Streptomyces caespitosus* broth. In addition to its antineoplastic effect, it also inhibits fibroblast proliferation (Attard and MacLean 2007). In this study, we evaluated the comparative effectiveness of MMC and HA/CMC for the prevention of IAs.

In a systematic review and meta-analysis, Zeng et al. (2007) addressed the efficacy and safety of HA/CMC. Their analysis concluded that HA/CMC could decrease abdominal adhesions after general surgery. However, they also reported that HA/CMC did not reduce postoperative intestinal obstructions and it increased abdominal abscess and anastomotic leak rates. In another study, Kumar et al. searched several databases in order to determine the efficacy and safety of several agents that were used to prevent IAs. They reported that the use of a HA/CMC membrane reduced the incidence, extent and severity of adhesions in re-operative abdominal surgery (Zeng et al. 2007). Burns et al (1997) studied HA/CMC for the prevention of IAs in an experimental model under ischemic conditions. They concluded that HA/CMC was safe and effective in reducing postsurgical adhesions. In a recent study, Caglayan et al. (2014) compared HA/CMC with ethyl pyruvate in rats. HA/CMC and ethyl pyruvate (EP) were found to reduce the formation IAs, however, no significant difference was found between HA/CMC and EP. In comparison, Stawicki et al. (2014) findings contrasted with other studies. The authors performed a prospective randomized controlled study. They reported that HA/CMC showed no favorable effects on the elimination of adhesion formation. In the present study, there was no significant reduction in inflammation, fibrosis and adhesion scores in the HA/CMC group when compared to the sham group.

MM-C was investigated by Tander et al., Cubukcu et al., and Liu et al. for the prevention of IAs in experimental models. All of these studies showed that MM-C effectively prevented the formation of adhesions (Ahmad et al. 2014; Tander et al. 2007; Liu et al. 2005). As mentioned above, the efficacy of MM-C has been well established in various clinical settings (Kaufman et al. 2013; Cheng et al. 2013; Cano-Parra et al. 1995; Mahindrakar et al. 2001; Minguini et al. 2000; Numthavaj et al. 2013; Daher et al. 2007; Lee et al. 2011; Nagaich et al. 2014). MM-C has become the agent of choice with favorable results in reducing postoperative scar formation. In the present study, MM-C showed no significant affect on inflammation scores. However, MM-C significantly reduced fibrosis scores and also showed a tendency towards the reduction of adhesion scores.

Phılıps et al. (1960) studied MM-C related toxic side effects in rats and mice. They reported that weight loss, bloody masks, diarrhea, etc., were evident within 4 days of application. In sternal bone marrow, cellularities were reduced in quantity and the reduction rates were 10–50 % at 9 days. They also described a lethal dose (LD50) of 2.5 mg/kg for MM-C, after a single or multiple intraperitoneal injections for 5 days. A limitation of the current study includes the fact that scarification was performed after 21 days of follow up. Thus, MM-C related transient complications in the hematopoietic system or on bone marrow cellularity may have been overlooked. However, the authors of the study have not observed or demonstrated any MMC related toxic or lethal effects. Another limitation of the study stems from the fact that the anti-adhesive affects of MM-C were not compared in different application doses. Thus, we were unable to assess the relationship between dose dependent anti-adhesive affects and toxic complications of MM-C.

## Conclusion

Despite its potential for systemic toxicity, Mitomycin-C shows some anti-fibrosis and anti-adhesive effects. MM-C is a promising agent for the prevention of IAs, and as such, further trials are warranted to study efficacy.
